# Diagnostic accuracy of 1p/19q codeletion tests in oligodendroglioma: A comprehensive meta‐analysis based on a Cochrane systematic review

**DOI:** 10.1111/nan.12790

**Published:** 2022-03-03

**Authors:** Sebastian Brandner, Alexandra McAleenan, Hayley E. Jones, Ashleigh Kernohan, Tomos Robinson, Lena Schmidt, Sarah Dawson, Claire Kelly, Emmelyn Spencer Leal, Claire L. Faulkner, Abigail Palmer, Christopher Wragg, Sarah Jefferies, Luke Vale, Julian P. T. Higgins, Kathreena M. Kurian

**Affiliations:** ^1^ Division of Neuropathology, The National Hospital for Neurology and Neurosurgery University College London Hospitals NHS Foundation Trust London UK; ^2^ Department of Neurodegenerative Disease, Queen Square Instituite of Neurology University College London London UK; ^3^ Population Health Sciences, Bristol Medical School University of Bristol Bristol UK; ^4^ Population Health Sciences Institute Newcastle University Newcastle upon Tyne UK; ^5^ Bristol Genetics Laboratory, Pathology Sciences Southmead Hospital Bristol UK; ^6^ Department of Oncology Addenbrooke's Hospital Cambridge UK; ^7^ Bristol Medical School: Brain Tumour Research Centre, Public Health Sciences University of Bristol Bristol UK

**Keywords:** 1p/19q codeletion, false negative, false positive, fluorescent in situ hybridisation, oligodendroglioma, PCR

## Abstract

Codeletion of chromosomal arms 1p and 19q, in conjunction with a mutation in the isocitrate dehydrogenase 1 or 2 gene, is the molecular diagnostic criterion for oligodendroglioma, IDH mutant and 1p/19q codeleted. 1p/19q codeletion is a diagnostic marker and allows prognostication and prediction of the best drug response within IDH‐mutant tumours. We performed a Cochrane review and simple economic analysis to establish the most sensitive, specific and cost‐effective techniques for determining 1p/19q codeletion status. Fluorescent in situ hybridisation (FISH) and polymerase chain reaction (PCR)‐based loss of heterozygosity (LOH) test methods were considered as reference standard. Most techniques (FISH, chromogenic in situ hybridisation [CISH], PCR, real‐time PCR, multiplex ligation‐dependent probe amplification [MLPA], single nucleotide polymorphism [SNP] array, comparative genomic hybridisation [CGH], array CGH, next‐generation sequencing [NGS], mass spectrometry and NanoString) showed good sensitivity (few false negatives) for detection of 1p/19q codeletions in glioma, irrespective of whether FISH or PCR‐based LOH was used as the reference standard. Both NGS and SNP array had a high specificity (fewer false positives) for 1p/19q codeletion when considered against FISH as the reference standard. Our findings suggest that G banding is not a suitable test for 1p/19q analysis. Within these limits, considering cost per diagnosis and using FISH as a reference, MLPA was marginally more cost‐effective than other tests, although these economic analyses were limited by the range of available parameters, time horizon and data from multiple healthcare organisations.

## Graphical abstract

In this summary of a Cochrane review, we analysed the diagnostic accuracy of 1p/19q codeletion tests in oligodendroglioma, to establish the most sensitive, specific and cost‐effective techniques. Fluorescent in situ hybridisation (FISH) and polymerase chain reaction (PCR)‐based loss of heterozygosity (LOH) test methods were considered as reference standards. Next‐generation sequencing and single nucleotide polymorphism arrays were found to have a high specificity. We found no difference in the hazard ratio for overall survival between studies, using two different techniques, PCR‐based LOH and FISH.

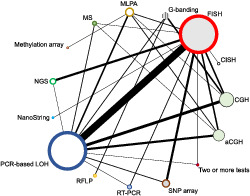

**Key Points**

- In a Cochrane review, we established the most sensitive, specific and cost‐effective techniques for determining 1p/19q codeletion status.
- Fluorescent in situ hybridisation (FISH) and polymerase chain reaction (PCR)‐based loss of heterozygosity (LOH) test methods were considered as reference standard.
- Next‐generation sequencing and single nucleotide polymorphism arrays have high specificity.
- No difference in the hazard ratio for overall survival was found between studies using two different techniques, PCR‐based LOH and FISH.

## INTRODUCTION

**FIGURE 1:**
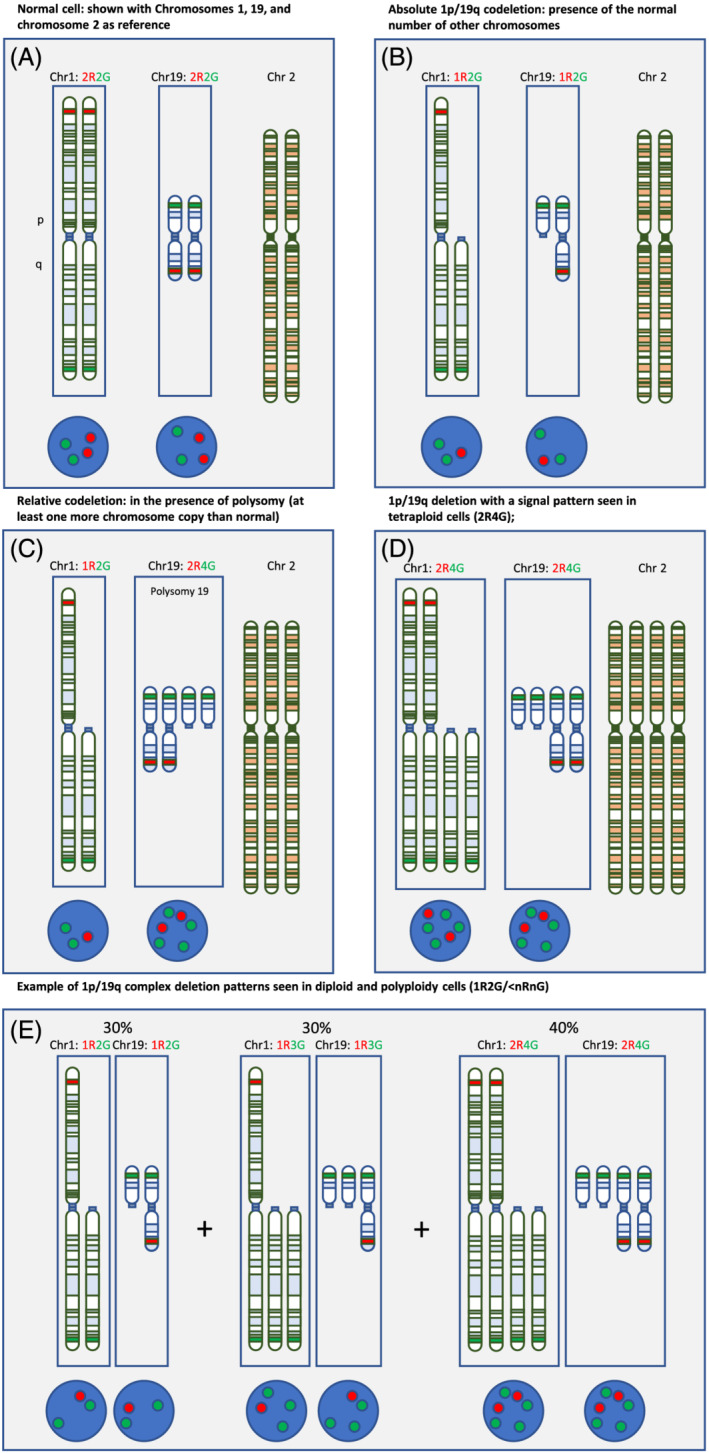
Graphical representation of absolute and relative 1p/19q codeletions. In all parts of the figure, chromosomes 1 and 19 are presented in separate frames to visualise the combination of FISH signals. The 1p and the 19q probes are red, and the reference probes (1q and 19p) are green. The approximate labelling sites are indicated in the chromosomal schematics. An unrelated chromosome (2) is also shown, and appearances as FISH images on the bottom of each frame. (A) Cell with diploid set of chromosomes, with two red signals each, for chromosomal arms 1p and 19q, as well as two green signals each for chromosomal arms 1q and 19p. (B) Absolute 1p/19q codeletion in a diploid set of chromosomes. Loss of one red signal in chromosome 1p and in 19q and two green signals for each 1q and 19p. (C) Relative codeletion with example of polysomy of chromosome 19 and chromosome 2, which has been suggested to indicate a worse prognosis [4, 5, 6, 7]. (D) 1p/19q codeletion in tetraploid cells, resulting in two red and four green signals for both 1p and 19q tests. (E) Complex deletion patterns as found in a small proportion of oligodendrogliomas, often associated with anaplastic histological types. In this example, there are diploid cells (left, 30%) triploid cells (centre, 30%) and tetraploid cells (right, 40%)

Complete deletion of both the short arm of chromosome 1 (1p) and the long arm of chromosome 19 (19q) (1p/19q codeletion) is a chromosomal alteration that occurs in oligodendrogliomas, but to date, the best method to detect such deletions is unclear. The codeletion is thought to be an early event in oligodendroglioma tumourigenesis [1] and is thought to be a result of an unbalanced whole‐arm translocation between chromosomes 1 and 19 with the loss of the resulting hybrid chromosome [2, 3] (Figure 1). The combined presence of an *IDH1* or *IDH2* mutation and a 1p/19q codeletion is a diagnostic criterion for oligodendroglioma, IDH mutant and 1p/19q codeleted [8]. The diagnostic test algorithm of IDH‐mutant gliomas has been streamlined in a recent consensus publication cIMPACt‐NOW update 5 [9], recommending that 1p/19q testing is not required in IDH‐mutant astrocytic tumours with loss of nuclear ATRX expression. Although this recommendation reduces the number of 1p/19q tests in IDH‐mutant gliomas, the diagnosis of oligodendroglioma, IDH mutant and 1p/19q codeleted, central nervous system (CNS) World Health Organization (WHO) Grade 2 or 3 still requires the detection of an IDH mutation and a 1p/19q codeletion. The European guidelines recommend that 1p/19q status is evaluated to support a diagnosis of oligodendroglioma, IDH mutant and 1p/19q codeleted, and for prognosis, and that treatment decisions are based on the 1p/19q status [10, 11, 12]. Current guidance from the National Institute for Health and Care Excellence (NICE) (United Kingdom) recommends and the 2021 CNS WHO classification [13] mandates testing 1p/19q codeletion to identify oligodendrogliomas, and the adjuvant chemotherapeutic recommended after surgery for people with CNS WHO Grade 3 glioma varies according to 1p/19q status (NICE 2018) [14].

1p/19q status can be determined by several different methods, and there is no consensus regarding the optimal method. The two most common methods for routine diagnostic use are FISH‐ and polymerase chain reaction (PCR)‐based loss of heterozygosity (LOH) assays [15]. In the 2017 UK Cytogenomic External Quality Assessment Service (CEQAS) report, of the 35 enrolled laboratories, 25 laboratories used FISH, 1 laboratory used multiplex ligation‐dependent probe amplification (MLPA), 4 laboratories used arrays and 1 laboratory used quantitative PCR.

Implementation and use of these techniques depend on infrastructure and economic circumstances of a country and of individual pathology departments. Therefore, the review considered the costs and the cost‐effectiveness of alternative methods assessing 1p/19q status. Each method incurs costs for laboratory, hospital occupancy and subsequent treatment. The benefits of targeted treatment may include greater survival and less exposure to potentially toxic treatments.

A recent systematic review and meta‐analysis of the prognostic value of chromosomal 1p/19q codeletion in CNS WHO Grade 2 and 3 oligodendrogliomas found a summary hazard ratio (HR) for mortality of 0.28 (95% confidence interval [CI] 0.13 to 0.62; 9 studies) favouring 1p/19q codeletion after adjusting for age, extent of resection, IDH mutation and type of therapy [16]. Another systematic review and meta‐analysis found that 1p/19q codeletion was associated with increased overall survival (HR 0.43; 95% CI 0.35–0.53; 14 studies) [17], both in WHO low‐grade (HR 0.45; 95% CI 0.30–0.68; 5 studies) and high‐grade oligodendrogliomas (HR 0.41; 95% CI 0.31–0.53; 6 studies), and for astrocytic tumours (HR 0.52; 95% CI 0.36–0.75; 3 studies) and oligodendroglial tumours (HR 0.41; 95% CI 0.30–0.56; 9 studies) [17]. This review also observed no evidence of difference in the HR for overall survival between studies using two different techniques (PCR‐based LOH and FISH) to assess the status of chromosomal arms 1p and 19q [17]. It is important to note that these studies were carried out before the current definition of oligodendroglioma, which now mandates the presence of an IDH mutation and a 1p/19q codeletion.

1p/19q codeletion can be absolute, that is, loss in the presence of the normal number of other chromosomes, or relative if it occurs in the presence of polysomy (when cells contain at least one more copy of a chromosome than normal) or polyploidy (when cells contain more than two sets of chromosomes) (Figure 1B–D). Several studies have suggested that people with relative 1p/19q codeletions (deletions in the presence of polysomy or polyploidy) have a worse prognosis (progression free survival or overall survival) than people with absolute 1p/19q codeletions, with some studies suggesting that prognosis in patients with relative codeletions may be similar to that of people with no codeletion at all [4, 5, 6, 18]. In all these studies, classification of polysomy occurred when more than 30% of nuclei had more than two 1q and 19p signals, as assessed by FISH (Figure 1E). Although there are limitations to these studies, for example, non‐standardised treatment, these findings suggest that diagnosing absolute deletions is more important. The Cochrane review focuses primarily on detection of absolute deletions and in diagnosing situations where one copy of 1p/19q has been lost and the other copy duplicated (also termed copy‐neutral LOH). Combinations of chromosomal deletions in oligodendrogliomas and the corresponding signals in FISH are presented in a schematic representation in Figure 1.

In addition to the significant clinical implications associated with the diagnostic accuracy of techniques to diagnose 1p/19q codeletion status in oligodendroglioma patients, there are also significant potential resource implications regarding the accuracy of the test. The estimated costs associated with clinical care for a patient with glioma ranged between US$ 4755 and US$ 42,907, with reported costs converted into 2013 US $ using an exchange rate based on purchasing power parities [19]. It was also estimated that 55% of these costs were attributable to chemotherapy drugs, chiefly temozolomide. If these therapies can be targeted at those patients who will obtain the greatest benefit, this will make better use of limited healthcare resources.

This review will assess the sensitivity and specificity of any DNA‐based techniques that can be used on tumour tissue to evaluate 1p/19q codeletion status directly involved when performing the different test methods. In addition, a cost‐effectiveness model was developed to equate costs against the diagnostic performance of each of the diagnostic test methods.

## METHODS

The protocol for the review was published in the Cochrane Database of Systematic Reviews [20], and the review was undertaken and reported following Cochrane's guidance (which is consistent with the Preferred Reporting Items for Systematic Reviews and Meta‐Analyses [PRISMA]) [21]. A more detailed account of the methods and results can be found in the full Cochrane publication [20].

### Study eligibility

We included cross‐sectional studies that use two or more tests to assess 1p/19q status in tumour tissue from the same set of people. Studies needed to present either raw data or classified results for patients for at least two tests. Studies that reported only on concordance of test results were not included. Studies with data for just one person were excluded. For the integrated review of economic evidence, we sought cost and full economic evaluations (cost‐effectiveness analyses, cost‐utility analyses and cost–benefit analyses) that had been conducted alongside any study designs or as part of a modelling exercise. Participants were adults (≥18 years old) with glioma. Studies in which participants were recruited on the basis of their 1p/19q codeletion status were excluded.

### Search methodology

Searches included MEDLINE Ovid (1946–2019), Embase Ovid (1974–2019) and BIOSIS Citation Index (1969–2019). No restriction of language or date of publication was applied. Further searches included OpenGrey (http://www.opengrey.eu/), dissertations and theses (ProQuest Dissertations & Theses Global [https://search.proquest.com/pqdtglobal/dissertations/]) and the Networked Digital Library of Theses and Dissertations (http://search.ndltd.org/index.php). Abstracts from meetings of the Society for Neuro‐Oncology (SNO) and its partner associations, the European Association of Neuro‐Oncology (EANO) and the Japan Society of Neuro‐Oncology, were searched via the Web of Science Conference Proceedings Citation Index (CPCI‐S) (1990–2019). We also searched for any ongoing studies via the WHO International Clinical Trials Registry Platform (ICTRP) (all available years to 2019). Further studies were identified from reference lists of included studies. For the integrated review of economic evidence, suitable studies were searched in MEDLINE and Embase, and the National Health Service (NHS) Economic Evaluation Database (EED).

### Study selection, data extraction and quality assessment

**FIGURE 2:**
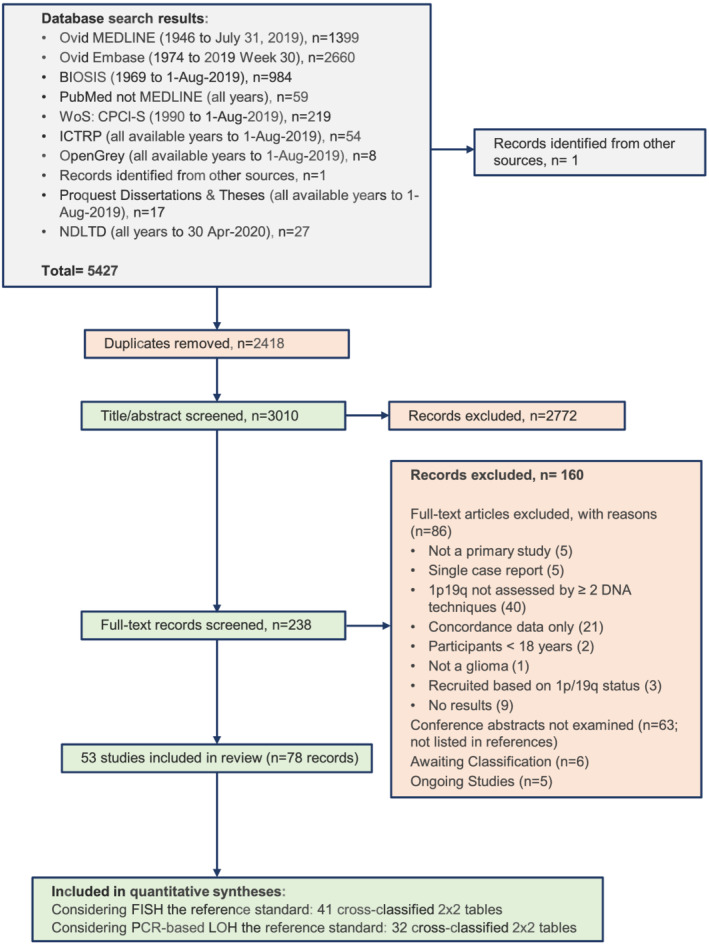
PRISMA flow chart illustrating the selection process of inclusions and exclusions of studies

We used EPPI‐Reviewer 4 (https://eppi.ioe.ac.uk) for processes of screening and selection of studies and for part of the data extraction [22]. Data were extracted and further analysed in Microsoft Excel. Two review authors (‘reviewers’) independently screened titles and abstracts of all identified search results and determined whether full texts should be retrieved. Then, two reviewers independently assessed the full‐text articles. Disagreements were resolved either by consensus or by consulting a third reviewer. A PRISMA [21] flow diagram was established to describe the flow of information through the different phases of the review (Figure 2).

Studies that met the inclusion criteria for diagnostic test accuracy (DTA) were screened by one reviewer to assess if any could possibly meet the economic inclusion criteria. Had any potentially relevant studies been identified, they would have been screened by two reviewers.

Two reviewers assessed risk of bias and applicability of the DTA studies using the QUADAS‐2 tool [23] tailored to our review. Disagreements were resolved by consensus, with discussion with a third review author if necessary.

### Index tests and target conditions

Studies using any DNA‐based technique to determine 1p/19q status in tumour tissue were included, whereas studies using immunohistochemically detection of 1p/19q status, or studies assessing 1p/19q status from blood samples of imaging, were excluded. The target condition was an absolute 1p/19q codeletion, that is, in the absence of polysomy. As described in Table 1, each of the tests can potentially generate false positive and false negative results. As such, there is no true ‘gold standard’ reference test and all tests are considered to be ‘index tests’. However, in order to estimate the sensitivity and specificity of each test, we considered two alternative reference standards: (i) using FISH as the reference standard, that is, assuming that FISH has 100% sensitivity and specificity, and (ii) using PCR‐based LOH assays as the reference standard, that is, assuming that PCR‐based LOH assays have 100% sensitivity and specificity. The use of FISH or PCR‐based LOH assays was not an inclusion criterion: All studies that used two or more tests to assess 1p/19q status in tumour tissue from the same set of people were included in the review.

**TABLE 1:** Summary of techniques with brief description and diagnostic accuracy of study results

Technique	Acronym	Description of the technique	Quality of the evidence (GRADE) reference standard: FISH	Participants (studies)	Sensitivity [95% CI]	Specificity	Codel FISH detected^a^	Correct positive^a^	False negative^a^	No codel FISH^a^	Correct negative^a^	False positive^a^	Quality of the evidence (GRADE) reference standard: PCR‐based LOH	Participants (studies)	Sensitivity [95% CI]	Specificity	Codel PCR detected^b^	Corr positive^b^	False negative^b^	No codel PCR^b^	Correct negative^b^	False positive^b^
Fluorescent in situ hybridisation	FISH	Fluorescently labelled probes hybridise to specific chromosomal regions. Can be performed on FFPE, preserves tissue architecture, assessed by fluorescent microscopy	Reference standard										Low certainty evidence: rated down due to risk of bias and indirectness	915 (15)	0.91 [0.78–0.97]	0.96 [0.90–0.99]	31	28	3	69	66	3
Chromogenic in situ hybridisation	CISH	Chromogenic‐labelled probes hybridise to specific chromosomal regions. Can be performed on FFPE, preserves tissue architecture, assessed by bright‐field microscopy	Low certainty evidence: rated down due to high imprecision	38 (1)	1.00 [0.84–1.00]	0.92 [0.33–1.00]	31	31	0	69	63	6	No results									
PCR‐based LOH assays (also known as PCR‐based short tandem repeat or microsatellite analysis)	PCR‐based LOH	Analysis of polymorphic microsatellites dispersed throughout the genome. Different alleles have different numbers of repeats, resulting in different length PCR products. PCR of these regions in individuals with two different alleles (heterozygous) results in two different length products. If heterozygosity is lost, only one length product will be obtained. An individual must be heterozygous for a microsatellite for it to be informative, and DNA from normal tissue is required to determine this. Primers that amplify regions containing microsatellites on 1p and 19q can be used to determine whether 1p and 19q are codeleted. No consensus exists on location or number of microsatellites analysed	Low certainty evidence: rated down due to risk of bias and indirectness	915 (15)	0.94 [0.83–0.98]	0.94 [0.87–0.98]	31	28	3	69	65	3	Reference standard									
Restriction fragment length polymorphism analysis	RFLP	Restriction enzymes recognising specific sequences cut genomic DNA into fragments of specific sizes. Different alleles may contain cut sites, or the DNA fragment that the restriction enzyme produces after digestion may be expected to differ due to different numbers of repeats in different alleles. Therefore, in a similar manner to PCR, LOH can be detected through loss of fragments of a specific size from informative loci (where an individual is heterozygous in normal tissue)	No results										No results									
Comparative quantitative PCR, real‐time PCR	qPCR	Comparison of PCR product (amplicon) from 1p/19q with amplicon obtained from other chromosomal regions. A deletion results in reduced amplicon, which can be quantified by comparison with the reference amplicon	Very low certainty evidence: rated down due to high risk of bias, high imprecision and indirectness	40 (2)	0.81 [0.20–0.99]	1.00 [0.95–1.00]	31	25	6	69	69	0	Very low certainty evidence: rated down due to risk of bias, imprecision and indirectness	10 (1)	1.00 [0.77–1.00]	NA^b^	31	31	0	—^b^	—^b^	—^b^
Multiplex ligation‐dependent probe amplification	MLPA	Use of ‘split’ probes containing that hybridise to specific genomic regions and also contain primer binding sites. Following hybridisation, a ligation step joins adjacent probes, which are subsequently amplified at the primer binding sites by PCR. Only ligated pairs will be amplified. PCR products are separated by length, quantified, normalised internally and compared with reference samples	Very low certainty evidence: rated down due to risk of bias, high imprecision and indirectness	33 (2)	0.96 [0.44–1.00]	0.68 [0.20–0.95]	31	30	1	69	47	22	Very low certainty evidence: rated down due to high risk of bias and indirectness	18 (1)	1.00 [0.74–1.00]	1.00 [0.83–1.00]	31	31	0	69	69	0
Comparative genomic hybridisation	CGH	Tumour and normal tissue genome (can be from different people) are differentially labelled with two different fluorochromes and then simultaneously hybridised to normal metaphase chromosomes. Chromosomal copy number changes alter the ratio of the two genomes, measured by differential intensities of the fluorochromes	Low certainty evidence: rated down due to risk of bias and imprecision	75 (4)	0.95 [0.59–1.00]	0.99 [0.90–1.00]	31	31	0	69	63	6	Low certainty evidence: rated down due to risk of bias and indirectness	151 (6)	0.94 [0.74–0.99]	0.98 [0.91–1.00]	31	29	2	69	68	1
Array CGH	aCGH	Same principles as CGH, but instead of the two genomes being competitively hybridised to metaphase chromosomes, they are hybridised to a microarray. The theoretical resolution of aCGH is greater than that of traditional CGH	Very low certainty evidence: rated down due to risk of bias, imprecision and indirectness	39 (3)	1.00 [0.89–1.00]	0.91 [0.55–0.99]	31	31	0	69	63	6	Low certainty evidence: rated down due to high risk of bias	57 (4)	1.00 [0.97–1.00]	0.96 [0.75–1.00]						
Single nucleotide polymorphism arrays	SNP array	DNA microarray to determine copy number and genotype, can detect gains, losses and copy‐neutral LOH. SNPs are variations at a single position in a DNA sequence, one copy of each SNP position inherited from each parent resulting in genotypes *AA*, *AB* or *BB*. To detect abnormalities using SNP arrays, sample DNA is fragmented, labelled and hybridised to an array containing immobilised allele‐specific oligonucleotide probes and signal intensity of individual probes measured and copy numbers are calculated	Very low certainty evidence: rated down due to risk of bias, imprecision and indirectness	111 (6)	0.90 [0.57–0.99]	0.97 [0.84–1.00]	31	28	3	69	67	2	Very low certainty evidence: rated down due to risk of bias and high imprecision	33 (2)	0.97 [0.50–1.00]	1.00 [0.92–1.00]	31	30	1	69	69	0
Methylation arrays		The main purpose of methylation arrays is the measurement of specific regions of the genome that may be modified by methylation and the methylation profile is compared with a reference set of tumours. The array has two probes for each region, one for the methylated and one for unmethylated. To detect copy number variations, the signals from both probes (the methylated and unmethylated) for a specific region are added together and compared with a reference genome, and these data can be used to detect chromosomal changes including 1p/19q status	No results										No results									
Next‐generation sequencing	NGS	NGS refers to post‐Sanger sequencing technologies including sequencing by synthesis, sequencing by ligation and ion semiconductor sequencing. Although traditional Sanger sequencing sequences a single‐DNA sequence, NGS is capable of sequencing multiple sequences simultaneously. Techniques have been developed to detect LOH and copy number variations using NGS. Deletions can be detected by relative perturbations in the read depth. LOH can be detected when the ratio of alleles at a heterozygous SNP site is perturbed	Low certainty evidence: rated down due to risk of bias and indirectness	243 (6)	0.94 [0.75–0.99]	1.00 [0.99–1.00]	31	29	2	69	69	0	Very low certainty evidence: rated down due to risk of bias, imprecision and indirectness	49 (1)	1.00 [0.86–1.00]	0.98 [0.64–1.00]	31	31	0	69	68	1

*Note*: Orange fields indicate reference standard. Blue fields indicate techniques used in studies and were compared to a reference standard. Grey fields indicate studies for which no reference standard was available.

^a^ Narrative for these fields: All hypothetical scenarios assume that 31 people out of 100 with glioma will have a FISH‐detected 1p/19q codeletion. Taking the example of CISH: Of these, 31 people will be given the correct positive result and 0 people will be given a false negative result; of the 69 people without the codeletion, 68 people will be given a correct negative result and 1 people will be given a false positive result.

^b^ Narrative for these fields: All hypothetical scenarios assume that 31 people out of 100 with glioma will have a PCR‐based LOH‐detected 1p/19q codeletion. Taking the example of NGS: Of these, 31 people will be given the correct positive result and 0 people will be given a false negative result. Of the 69 people without the codeletion, 68 people will be given a correct negative result and 1 people will be given a false positive result.

### Statistical analysis and data synthesis

For analysis with each of the respective reference standards (FISH or PCR‐based LOH tests), we performed bivariate meta‐analyses of the sensitivity and false positive rate (1−specificity) of each index test, assuming binomial likelihoods for the number of ‘true positive’ and ‘true negative’ test results (2 × 2 table) [24, 25]. This approach allows for heterogeneity in sensitivity and specificity across studies and for between‐study correlation in these measures. In our main analyses, we assumed that this between‐study correlation and the standard deviation (heterogeneity) parameters were shared (i.e., identical) across tests. For studies comparing more than one test with the reference standard, multiple 2 × 2 tables were derived.

### Economic model: Base‐case analysis

In addition to the clinical analysis of the results, this study includes a model‐based cost‐effectiveness analysis to compare the costs and diagnostic performance of the different tests. The model is a mathematical framework that can be used to estimate the consequences of healthcare decisions [26]. This model took the form of a decision tree, and the time horizon for this model was until diagnosis. As such, this model does not include costs and health outcomes beyond diagnosis.

The data required for the model included the prevalence of glioma, the sensitivity and specificity of the tests, and the cost of providing the tests. Prevalence of glioma was derived from the results of the meta‐analysis. The sensitivity and specificity values that were calculated in the meta‐analysis were utilised in the decision model. For the cost values, intervention costs were derived from both expert opinions from within the Newcastle upon Tyne Hospitals NHS Foundation Trust based on internal costings (costs for FISH and chromogenic in situ hybridisation [CISH], real‐time PCR and PCR‐based LOH, MLPA and single nucleotide polymorphism [SNP] array), whereas cost for next‐generation sequencing (NGS) and array CGH (aCGH) were derived from existing literature [27, 28]. These costs were then checked for face validity with other members of the review team with experience of the provision. All costs are reported in 2020 Great Britain pound sterling (GBP), and where necessary cost were converted into 2020 GBP using the EPPI‐Centre Cost Converter [29]. No cost for the G banding, karyotyping, mass spectrometry (MS) and NanoString techniques and comparative genomic hybridisation (CGH) was identified, as they are currently not routinely performed in the UK NHS and thus were not included. The model parameters are presented in Tables S1 and S2. The model was designed to generate the expected costs per true positive diagnosis, per true negative diagnosis and per correct diagnosis.

### Economic model: Sensitivity analysis

A probabilistic sensitivity analysis (PSA) was carried out to address the uncertainty around the conclusions of the economic model. A PSA allows uncertainty caused by the imprecision surrounding the estimates used in the model examined simultaneously. Therefore, for each model parameter, a distribution was defined. A triangular distribution was used for cost values, and beta distributions were used for the prevalence, sensitivity and specificity values. Monte Carlo simulation was used to derive a distribution for cost and cost‐effectiveness. In the Monte Carlo simulation, a set of parameter values is then drawn by randomly sampling from each distribution. For each iteration of model parameters, the model outputs were estimated. This sampling process was repeated 10,000 times to produce distributions for each of the specified model outputs.

### Deviation from protocol

We had planned to perform a latent class analysis of all available data. We did not do this due to the complex structure of the data (with multiple studies involving different selections of test and different numbers of tests), which would involve development and validation of novel statistical methods. However, results from a limited latent class analysis of just FISH and PCR‐based LOH are reported in the full Cochrane review.

## RESULTS

### Search results and included studies

Using the search methodology (Section 2), 5427 records were identified, and after removing duplicates, 3010 records were screened at title and abstract; 237 records were selected for full‐text review, and 53 studies (in 78 publications) met the inclusion criteria. Assessments of risk of bias were mixed, due largely to lack of information about procedures in the study reports. The main issue of applicability was that many studies included only patients with specific subtypes of glioma.

### Presentation of study findings

**FIGURE 3:**
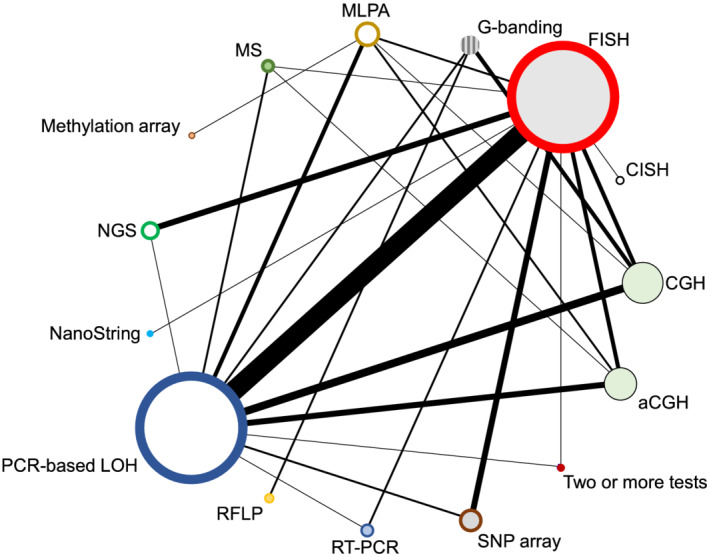
Network plot of the included studies. The colour scheme of the circles corresponds to the colour scheme of the test methods represented in Figures 4, 5, 6, 7. The size of the circles represents the number of test results for a test category. The thickness of the lines is proportional to the number of studies making the comparison. Note that the FISH and PCR‐based LOH circles include within‐test category comparisons

**FIGURE 4:**
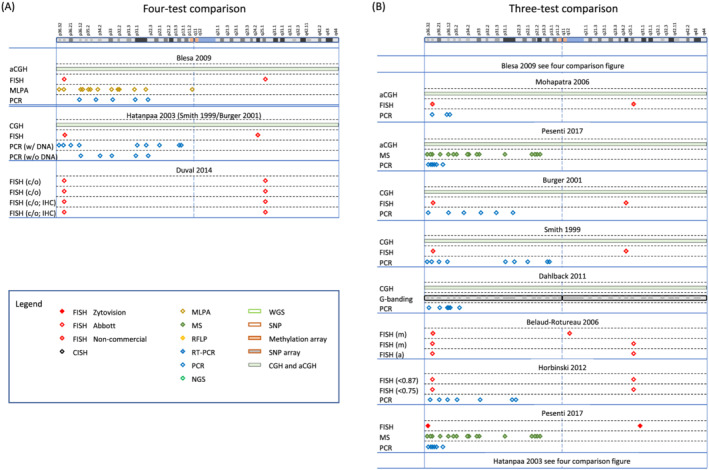
(A) Graphical representation of regions analysed in studies comparing four tests: Blesa 2009 [30], Hatanpaa 2003 [31] and Duval 2014 [32], and (B) studies comparing three tests: Mohapatra 2006 [33], Pesenti 2017 [34], Burger 2001 [35], Smith 1999 [36], Dahlback 2011 [37], Belaud‐Rotureau 2006 [38], Horbinski 2012 [39] and Pesenti 2017 [34]. The top on both figures indicates a graphical representation of chromosome 1 (adapted from the GRCh38/hg38 Assembly). The figure legend indicates the different methods, with different colour codes for FISH, depending on the origin or manufacturer of the probes. In each section, the first author of the study is represented on top, and the techniques on the left of the table. All acronyms are explained in the main text

**FIGURE 5:**
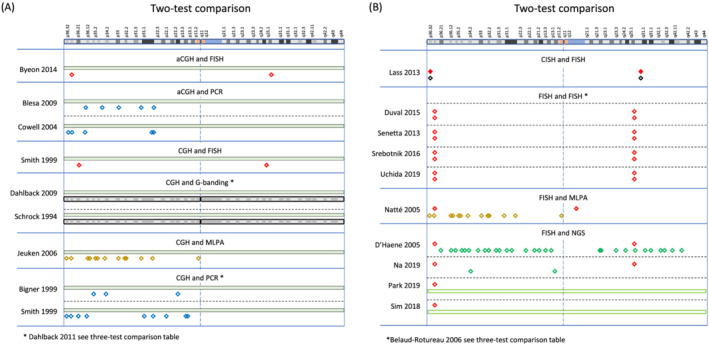
(A) Graphical representation of regions analysed in studies comparing two tests: aCGH and FISH (Byeon 2014 [40]), aCGH and PCR (Blesa 2009 [30] and Byeon 2014 [40]), CGH and FISH (Smith 1999 [36]), CGH and G banding (Dahlback 2009 [41] and Schrock 1994 [42]), CGH and MLPA (Jeuken 2006 [43]), and CGH and PCR (Bigner 1999 [44] and Smith 1999 [36]), and (B) CISH and FISH (Lass 2013 [45]), FISH and FISH (Duval 2015 [46], Senetta 2013 [47], Srebotnik‐Kirbis 2016 [48] and Uchida 2019 [49]), FISH and MLPA (Natté 2005 [50]), and FISH and NGS (D'Haene [51], Na 2019 [52], Park 2019 [53] and Sim 2018 [54]). The top of the figure indicates a graphical representation of chromosome 1 (adapted from the GRCh38/hg38 Assembly). For legend to symbols, see Figure 4

**FIGURE 6:**
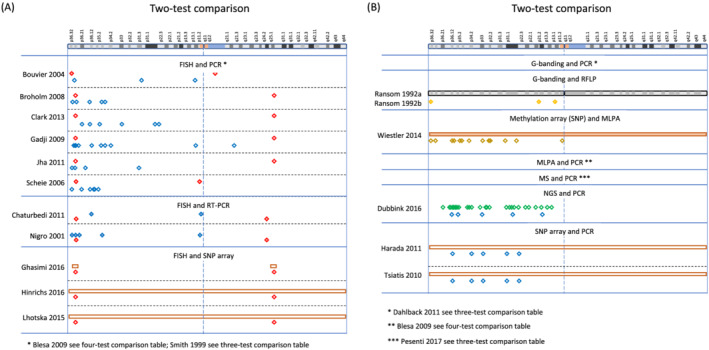
(A) Graphical representation of regions analysed in studies comparing two tests: FISH and PCR (Bouvier 2004 [55], Broholm 2008 [56], Clark 2013 [57], Gadji 2009 [58], Jha 2011 [59] and Scheie 2006 [60]), FISH and real‐time PCR (Chaturbedi 2011 [61] and Nigro 2001 [62]), and FISH and SNP array (Ghasimi 2016 [63], Hinrichs 2016 [64] and Lhotska 2015 [65]), and (B) G banding and RFLP (Ransom 1992 [66] and Ransom 1992 [67]), methylation array (SNP readout) and MLPA (Wiestler 2014 [68]), NGS and PCR (Dubbink 2016 [69]), and SNP array and PCR (Harada 2011 [70] and Tsiatis 2010 [71]). The top of the figure indicates a graphical representation of chromosome 1 (adapted from the GRCh38/hg38 Assembly). For legend to symbols, see Figure 4

**FIGURE 7:**
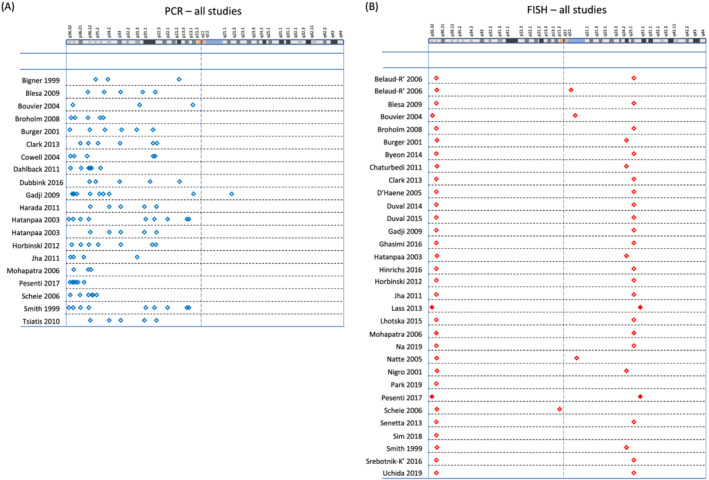
(A) Graphical representation of PCR primer locations used in studies comparing PCR with other methods. Studies appear in alphabetical order of first author: Bigner 1999 [44], Blesa 2009 [30], Bouvier 2004 [55], Broholm 2008 [56], Burger 2001 [35], Clark 2013 [57], Cowell 2004 [72], Dahlback 2011 [37], Dubbink 2016 [69], Gadji 2009 [58], Harada 2011 [70], Hatanpaa 2003 [31], Horbinski 2012 [39], Jha 2011 [59], Mohapatra 2006 [33], Pesenti 2017 [34], Scheie 2006 [60], Smith 1999 [36] and Tsiatis 2010 [71]. (B) Graphical representation of FISH probe locations used in studies comparing FISH with other methods: Belaud‐Rotureau 2006 [38], Blesa 2009 [30], Bouvier 2004 [55], Broholm 2008 [56], Burger 2001 [35], Byeon 2014 [40], Chaturbedi 2011 [61], Clark 2013 [57], D'Haene 2019 [51], Duval 2014 [32], Duval 2015 [46], Gadji 2009 [58], Ghasimi 2016 [63], Hatanpaa 2003 [31], Hinrichs 2016 [64], Horbinski 2012 [39], Jha 2011 [59], Lass 2013 [45], Lhotska 2015 [65], Mohapatra 2006 [33], Na 2019 [52], Natté 2005 [50], Nigro 2001 [62], Park 2019 [53], Pesenti 2017 [34], Scheie 2006 [60], Senetta 2013 [47], Sim 2018 [54], Smith 1999 [36], Srebotnik‐Kirbis 2016 [48] and Uchida 2019 [49]. The top of the figure indicates a graphical representation of chromosome 1 (adapted from the GRCh38/hg38 Assembly). For legend to symbols, see Figure 4

A network plot illustrates comparisons of test methods that were made among the included studies (Figure 3). A summary of the study findings and meta‐analysis results is presented in Table 1. Tests that are relevant in clinical practice (PCR‐based LOH, FISH, aCGH, SNP array, NGS, MLPA and real‐time PCR) are shown with a brief explanation of the technique, and each test has been compared with one of the two index tests, FISH and PCR‐based LOH, with separate listing of the quality of evidence, number of participants in the study, sensitivity, specificity, and an explanatory indication of the sensitivity and specificity, putting into a more intuitive context how many people with a positive test result achieved with index test (‘codeletion FISH detected’ or ‘codeletion PCR detected’) will have a correct positive, or a false positive result, and how many people with a detected non‐codeletion have a correct negative or a false negative result. The table indicates the outcome from the assessment using the GRADE approach [73, 74] with certainty of evidence (‘high’, ‘moderate’, ‘low’ or ‘very low’), considering risk of bias, imprecision, inconsistency, indirectness and publication bias, all of which may lead to downgrading the quality of the evidence. All tests performed are also graphically represented in Figures 4, 5, 6, 7.

### Comparison of studies with FISH as reference standard

From the included studies that performed FISH (Figure 7B) and at least one other test that was not a FISH variant, we created 41 cross‐classified 2 × 2 tables, with FISH as the reference standard (Table 1). FISH was compared with 10 different test categories: PCR‐based LOH (15 comparisons), SNP array (6), NGS (6), CGH (4), aCGH (3), MLPA (2), real‐time PCR (2), CISH (1), MS (1) and NanoString (1). The forty‐one 2 × 2 tables came from 33 studies: 26 studies compared FISH with one other test, 6 studies compared FISH with two other test categories and 1 study compared FISH with three other test categories (Figures 4, 5, 6, 7). The main results from the bivariate meta‐analysis model indicate that sensitivity and specificity were generally high, though with wide credible intervals for most tests, and some results are based on very small numbers of patients, such as the result for mass spectrometry, which is based on a single study of 10 people. Our GRADE assessments for all tests were either ‘low’ or ‘very low’.

### Comparison of studies with PCR‐based LOH as reference standard

From the included studies that performed PCR‐based LOH (Figure 7B) and at least one other test that was not a PCR‐based LOH variant, we created 32 cross‐classified 2 × 2 tables, treating PCR‐based LOH as a reference standard (Table 1). PCR‐based LOH was compared with nine different test categories: FISH (15 comparisons), CGH (6), aCGH (4), SNP array (2) and NGS, G banding, MLPA, real‐time PCR and MS (1 each). The thirty‐two 2 × 2 tables came from 22 studies: 14 studies compared PCR‐based LOH with one other test, 6 studies compared PCR‐based LOH with two other test categories and 2 studies compared PCR‐based LOH with three other test categories (Figures 4, 5, 6, 7). Results from the main bivariate meta‐analyses are again based on very low numbers of patients. A poor estimate of sensitivity for G banding/karyotyping is based on a single study in which none of 13 PCR‐detected 1p/19q codeletions were identified. Our GRADE assessments for all tests were either ‘low’ or ‘very low’.

### Results from economic model

The results for the base case of the economic model are summarised in Table S1 (FISH as reference standard) and Table S2 (PCR‐based LOH as reference standard). A prevalence of 0.31 was used on the basis of the included studies in the meta‐analysis. For several of the techniques in the meta‐analysis, there is a smaller number of studies or participants, and therefore, the point estimates in Tables S1 and S2 must be interpreted with caution.

### Sensitivity analysis

The results of the sensitivity analysis are displayed in Tables S1 and S2. The cost‐effectiveness was compared with a number of different thresholds of societal willingness to pay (WTP) for the three outcomes: (i) cost per true positive, (ii) cost per true negative or (iii) cost per case detected. These thresholds ranged from GBP (£) 0 (i.e., the decision is made on cost alone, and the test with the lowest cost would always be considered the most cost‐effective) to £10,000 (i.e., the amount willing to be paid for an additional unit of effect such as an additional true positive detected). When considering FISH as the reference standard, MLPA is the most likely to be considered cost‐effective. The other tests do have greater likelihood becoming cost‐effective at higher financial thresholds, but MLPA remains the most likely to be cost‐effective. The same is true for cost per true negative: For correct diagnosis, real‐time PCR had the highest probability of being cost‐effective at a WTP of £500 and £1000, and aCGH had the highest probability of being cost‐effective at a WTP of £5000 and £10,000. However, none of the seven tests compared over a range of thresholds had a probability of test being cost‐effective over 60%. By comparison, when PCR‐LOH was used as the reference standard, MLPA had a 100% probability of being considered the least costly of the five tests. MLPA also had the highest probability of being cost‐effective in terms of true positives, true negative and correct diagnoses at a WTP up to £10,000. However, at £5000 and £10,000, no test had a probability of being cost‐effective above 55%.

## DISCUSSION

To our knowledge, this is the first systematic review of the DTA of different techniques for assessing 1p/19q codeletion in glioma. We undertook a thorough search, applied systematic methods and assessed results for risk of bias using the QUADAS‐2 tool.

The systematic review found that most techniques, except G banding, have a very good sensitivity when comparing with FISH or PCR‐based LOH assay. G banding has a low sensitivity and specificity, but is no longer in routine laboratory use. Mass spectroscopy has a high sensitivity and specificity based on comparison with FISH and PCR‐based LOH, but data are based on a single study and the technology is not used in clinical diagnostic use. NGS and SNP array had high specificity when compared against FISH and also PCR‐based LOH, which is expected as these techniques determine entire chromosomal arms. This is of clinical importance, as in particular NGS is an expanding technology and increasingly used in diagnostic services. Whilst SNP arrays as such are rarely used nowadays, SNP data are determined from DNA methylation arrays, which are commonly used in brain tumour diagnostics [75, 76, 77, 78, 79, 80]. The illustration in Figure 1 shows the location of probes on the 1p chromosome. Our test accuracy results confirm previous studies [17], showing that there is no difference in the HR for overall survival between studies using two different techniques, PCR‐based LOH and FISH.

A technique of increasing importance is methylation array profiling. It is primarily used to establish epigenetic profiles of brain tumours, but the array data also generate a copy number profile with the added benefit of visualising chromosomal aberrations including 1p/19q codeletion [75, 76, 78, 79, 80]. This has been reported in two comparative studies [81, 82].

The limitation of our evaluation is the analysis of studies with FISH and PCR‐based LOH as reference standards, and none of the investigated tests can be found to be superior to the reference standard assumed. Consequentially, we were unable to include the results of studies that did not investigate either FISH or PCR‐based LOH in the statistical synthesis. Most studies did not distinguish between absolute and relative deletions, and even if technically possible, it was rarely reported. Furthermore, loss of 1p and 19q in combination with 1q and/or 19p was considered by some studies to count as 1p/19q codeletion, but not in others. When we had to interpret the results of techniques, we did so by looking for the presence/absence of 1p and 19q without consideration of 1q and 19p.

The current definition of oligodendrogliomas requires the presence of an IDH mutation combined with a 1p/19q codeletion [13], and therefore, the inclusion criteria of previous studies and clinical trials, when based on histological diagnosis, would not be valid nowadays. Therefore, statements such as ‘1p/19q codeletion allow for prognostication and prediction of the best drug response’ have to be viewed also in historical context. However, this statement could still be considered as adequate in the context of IDH‐mutant tumours as the 1p/19q codeletion delineates oligodendrogliomas from IDH‐mutant astrocytomas.

This economic decision model was the first to consider the costs and benefits of diagnostic test methods of identifying 1p and 19q status. When evaluating the results generated by the economic model, it is important to consider its limitations. One such limitation is around certain model inputs, some of which were derived from a single hospital estimate (laboratory costs, though we did conduct some checks on the face validity of these estimates) or study (some sensitivity and specificity estimates). To address this, distributions were attached to parameter estimates as part of the PSA, but future research could provide a wider range of real‐world parameters to explore the certainty of the model conclusions. Another limitation of this model is the limited time horizon. The model at present includes healthcare costs to derive the diagnosis of 1p and 19q status. However, assessing the long‐term costs and consequences of the diagnosis could have significant resource implications. For example, the costs and health impacts of outcomes such as a correct diagnosis or impacts of a false negative are not included in this model. Future research could focus on the long‐term implications of diagnosis of 1p/19q status and the impacts on treatment and health outcomes. This would allow testing strategies to be more fully evaluated and inform future decisions regarding diagnostic techniques.

Another parameter of practical importance, which could not be explored in the review is the time required to perform tests, which can indirectly impact cost‐effectiveness. Depending on the laboratory setting, PCR‐based tests for codeletion are considered less time‐consuming than FISH [83], in particular when performed alongside other tests requiring DNA extraction (*IDH1* and *IDH2* sequencing, and *MGMT* promoter methylation), whereas another study has reported no difference of turnaround time between FISH and real‐time reverse transcription PCR [84]. FISH may still be time effective in small‐volume settings and where dedicated technical staff are readily available. NGS approaches or methylation arrays require batching of samples and are currently less time effective, perhaps with the exception in high‐volume services. These techniques however offer a such significant additional information content, often allowing a conclusive diagnosis in a single assay, that this can compensate for the longer turnaround. Novel technologies, such as nanopore sequencing, are emerging, and these could significantly reduce testing times [85, 86].

In conclusion, the diagnosis of oligodendroglioma, IDH mutant and 1p/19q codeleted requires the demonstration of 1p/19q codeletion [10, 11, 87], but there is little consensus regarding the best approach. Our review suggests that all techniques except G banding have high sensitivity when compared against FISH or PCR‐based LOH as a reference standard, with NGS and SNP array having high specificity against FISH and PCR‐based LOH. This suggests that NGS and SNP array techniques can be used with confidence for detection of 1p/19q codeletion in the place of FISH or PCR‐based LOH, which may be advantageous as these techniques are capable of simultaneously detecting other abnormalities. The use of methylation arrays for brain tumour diagnostics is a recent development. The copy number information (equivalent to SNP data) of the arrays generates a diagnostic readout in addition to the high diagnostic value of a methylation class as described in the Cochrane review. The results of an accompanying economic model highlight potentially promising strategies for future research, but these results are compounded by a lack of data to parameterise the model and a limited time horizon. Future research can focus on deriving more longitudinal data to inform future economic evaluation studies assessing the long‐term health costs and consequences of such strategies.

## AUTHOR CONTRIBUTIONS

A. M., L. S., S. D., E. S. L. and C. L. F. performed the title/abstract screening. A. M., L. S., E. S. L. and J. P. T. H. performed the full‐text screening. A. M., L. S., C. K., E. S. L., A. P., J. P. T. H. and K. M. K. performed the data extraction. S. D. undertook the searches. A. M., L. S., C. K., J. P. T. H. and K. M. K. undertook the QUADAS‐2 assessments. H. E. J. performed the statistical analyses. A. K. and T. R. performed the economic analyses. J. P. T. H. performed the GRADE assessments. A. M. managed the project. J. P. T. H., L. V. and H. E. J. provided the methodological expertise. K. M. K., S. B., C. L. F. and C. W. provided the content expertise. A. M., H. E. J., A. K., T. R., S. B., L. V., J. P. T. H. and K. M. K. drafted the manuscript. All authors commented on the manuscript.

## CONFLICT OF INTERESTS

The authors declare no conflicts of interest.

### PEER REVIEW

The peer review history for this article is available at https://publons.com/publon/10.1111/nan.12790.

## Supporting information


**Table S1.** Costs and diagnostic accuracy of diagnostic tests to evaluate 1p/19q status codeletion (FISH as reference standard)
**Table S2.** Costs and diagnostic accuracy of diagnostic tests to evaluate 1p/19q status codeletion (PCR‐LOH as reference standard)

## Data Availability

Data sharing is not applicable to this article as no new data were created or analysed in this study.
