# Supplementary material for: Diagnostic accuracy of 1p/19q codeletion tests in oligodendroglioma: A comprehensive meta‐analysis based on a Cochrane systematic review
Source: Neuropathol Appl Neurobiol. 2022 Mar 3;48(4):e12790. doi: 10.1111/nan.12790 (PMC9208578; doi:10.1111/nan.12790)
Supplement: Supplementary file 1 — Table S1. Costs and diagnostic accuracy of diagnostic tests to evaluate 1p/19q status codeletion (FISH as reference standard) Table S2. Costs and diagnostic accuracy of diagnostic tests to evaluate 1p/19q status codeletion (PCR‐LOH as reference standard) [file NAN-48-e12790-s001.docx]

| **Supplementary Table 1 – Costs and diagnostic accuracy of diagnostic tests to evaluate 1p/19q status codeletion (FISH as reference standard)** | | | | | | | | | | |
| --- | --- | --- | --- | --- | --- | --- | --- | --- | --- | --- |
| **Incremental Cost Per True Positive Detected** | | | | | | | | | | |
| **Inputs** | | | **Deterministic Analysis** | | | **Probabilistic Sensitivity Analysis** | | | | |
| **Diagnostic Test** | **Cost (£)** | **Effect Rate** | **Incremental**  **Cost (£)** | **Incremental**  **Effect** | **ICER (£)** | **Prob of being CE at WTP of £0 per TP** | **Prob of being CE at WTP of £500 per TP** | **Prob of being CE at WTP of £1,000 per TP** | **Prob of being CE at WTP of £5,000 per TP** | **Prob of being CE at WTP of £10,000 per TP** |
| MLPA | 73 | 0.27 | - | - | - | 100% | 100% | 95% | 46% | 26% |
| PCR-Based LOH | 142 | 0.25 | - | - | Abs Dominated | 0% | 0% | 0% | 0% | 0% |
| RT-PCR | 142 | 0.24 | - | - | Abs Dominated | 0% | 0% | 2% | 2% | 1% |
| CISH | 186 | 0.30 | 113 | 0.03 | 3827 | 0% | 0% | 3% | 39% | 48% |
| Array CGH | 233 | 0.29 | - | - | Abs Dominated | 0% | 0% | 0% | 13% | 25% |
| SNP Array | 257 | 0.23 | - | - | Abs Dominated | 0% | 0% | 0% | 0% | 0% |
| NGS | 571 | 0.28 | - | - | Abs Dominated | 0% | 0% | 0% | 0% | 0% |
|  |  |  |  |  |  |  |  |  |  |  |
| **Incremental Cost Per True Negative Detected** | | | | | |  |  |  |  |  |
| **Diagnostic Test** | **Cost (£)** | **Effect Rate** | **Incremental**  **Cost (£)** | **Incremental**  **Effect** | **ICER (£)** | **Prob of being CE at WTP of £0 per TN** | **Prob of being CE at WTP of £500 per TN** | **Prob of being CE at WTP of £1,000 per TN** | **Prob of being CE at WTP of £5,000 per TN** | **Prob of being CE at WTP of £10,000 per TN** |
| MLPA | 73 | 0.45 | - | - | - | 100% | 16% | 1% | 0% | 0% |
| PCR-Based LOH | 142 | 0.65 |  | - | Abs Dominated | 0% | 30% | 28% | 13% | 7% |
| RT-PCR | 142 | 0.66 | 69 | 0.22 | 326 | 0% | 54% | 71% | 73% | 67% |
| CISH | 186 | 0.59 | - | - | Abs Dominated | 0% | 0% | 0% | 2% | 2% |
| Array CGH | 233 | 0.66 | - | - | Abs Dominated | 0% | 0% | 0% | 9% | 15% |
| SNP Array | 257 | 0.66 | - | - | Abs Dominated | 0% | 0% | 0% | 3% | 5% |
| NGS | 571 | 0.69 | 498 | 0.24 | 2111 | 0% | 0% | 0% | 0% | 4% |
|  |  |  |  |  |  |  |  |  |  |  |
| **Incremental Cost Per Correct Diagnosis** | | | | | |  |  |  |  |  |
| **Diagnostic Test** | **Cost (£)** | **Effect Rate** | **Incremental**  **Cost (£)** | **Incremental**  **Effect** | **ICER (£)** | **Prob of being CE at WTP of £0 per CD** | **Prob of being CE at WTP of £500 per CD** | **Prob of being CE at WTP of £1,000 per CD** | **Prob of being CE at WTP of £5,000 per CD** | **Prob of being CE at WTP of £10,000 per CD** |
| MLPA | 73 | 0.72 | - | - | - | 100% | 23% | 4% | 0% | 0% |
| PCR-Based LOH | 142 | 0.90 | - | - | Abs Dominated | 0% | 27% | 24% | 2% | 0% |
| RT-PCR | 142 | 0.91 | 69 | 0.19 | 362 | 0% | 47% | 54% | 27% | 18% |
| CISH | 186 | 0.89 | - | - | Abs Dominated | 0% | 3% | 10% | 12% | 9% |
| Array CGH | 233 | 0.95 | 160 | 0.24 | 673 | 0% | 0% | 8 | 58% | 60% |
| SNP Array | 257 | 0.89 | - | - | Abs Dominated | 0% | 0% | 0% | 0% | 0% |
| NGS | 571 | 0.97 | 498 | 0.25 | 1968 | 0% | 0% | 0% | 1% | 13% |

Abbreviations: WTP, willingness to pay; CE, cost effectiveness; TP, true positive; TN, true negative; CD, case detected; ICER incremental cost-effect ratio, MLPA, Multiplex ligation probe amplification; PCR, polymerase chain reaction; RT-PCR, real-time PCR; CISH, chromogenic in situ hybridisation; SNP, single nucleotide polymorphism; NGS, next generation sequencing.

| **Supplementary Table 2 – Costs and diagnostic accuracy of diagnostic tests to evaluate 1p/19q status codeletion (PCR-LOH as reference standard)** | | | | | | | | | | |
| --- | --- | --- | --- | --- | --- | --- | --- | --- | --- | --- |
| **Incremental Cost Per True Positive Detected** | | | | | | | | | | |
| **Inputs** | | | **Deterministic Analysis** | | | **Probabilistic Sensitivity Analysis** | | | | |
| **Diagnostic Test** | **Cost (£)** | **Effect Rate** | **Incremental**  **Cost (£)** | **Incremental**  **Effect** | **ICER (£)** | **Prob of being CE at WTP of £0 per TP** | **Prob of being CE at WTP of £500 per TP** | **Prob of being CE at WTP of £1,000 per TP** | **Prob of being CE at WTP of £5,000 per TP** | **Prob of being CE at WTP of £10,000 per TP** |
| MLPA | 73 | 0.28 | - | - | - | 100% | 99% | 94% | 61% | 45% |
| RT-PCR | 142 | 0.28 | - | - | Abs Dominated | 0% | 1% | 6% | 27% | 29% |
| FISH | 186 | 0.26 | - | - | Abs Dominated | 0% | 0% | 0% | 0% | 0% |
| Array CGH | 233 | 0.30 | 160 | 0.02 | 7507 | 0% | 0% | 0% | 12% | 24% |
| SNP Array | 257 | 0.28 | - | - | Abs Dominated | 0% | 0% | 0% | 0% | 2% |
| NGS | 571 | 0.29 | - | - | Abs Dominated | 0% | 0% | 0% | 0% | 0% |
|  |  |  |  |  |  |  |  |  |  |  |
| **Incremental Cost Per True Negative Detected** | | | | | |  |  |  |  |  |
| **Diagnostic Test** | **Cost (£)** | **Effect Rate** | **Incremental**  **Cost (£)** | **Incremental**  **Effect** | **ICER (£)** | **Prob of being CE at WTP of £0 per TN** | **Prob of being CE at WTP of £500 per TN** | **Prob of being CE at WTP of £1,000 per TN** | **Prob of being CE at WTP of £5,000 per TN** | **Prob of being CE at WTP of £10,000 per TN** |
| MLPA | 73 | 0.63 | - | - | - | 100% | 99% | 94% | 61% | 49% |
| FISH | 186 | 0.64 | - | - | Ext Dominated | 0% | 1% | 6% | 10% | 9% |
| Array CGH | 233 | 0.62 | - | - | Abs Dominated | 0% | 0% | 0% | 5% | 6% |
| SNP Array | 257 | 0.65 | 184 | 0.02 | 8686 | 0% | 0% | 0% | 24% | 35% |
| NGS | 571 | 0.65 | - | - | Abs Dominated | 0% | 0% | 0% | 0% | 1% |
|  |  |  |  |  |  |  |  |  |  |  |
| **Incremental Cost Per Correct Diagnosis** | | | | | |  |  |  |  |  |
| **Diagnostic Test** | **Cost (£)** | **Effect Rate** | **Incremental**  **Cost (£)** | **Incremental**  **Effect** | **ICER (£)** | **Prob of being CE at WTP of £0 per CD** | **Prob of being CE at WTP of £500 per CD** | **Prob of being CE at WTP of £1,000 per CD** | **Prob of being CE at WTP of £5,000 per CD** | **Prob of being CE at WTP of £10,000 per CD** |
| MLPA | 73 | 0.91 | - | - | - | 100% | 99% | 93% | 55% | 42% |
| FISH | 186 | 0.90 | - | - | Abs Dominated | 0% | 1% | 4% | 3% | 2% |
| Array CGH | 233 | 0.92 | - | - | Ext Dominated | 0% | 0% | 2% | 18% | 20% |
| SNP Array | 257 | 0.93 | 0.02 | 184 | 10372 | 0% | 0% | 1% | 24% | 29% |
| NGS | 571 | 0.94 | 0.03 | 498 | 15971 | 0% | 0% | 0% | 0% | 7% |

Abbreviations: WTP, willingness to pay; CE, cost effectiveness; TP, true positive; TN, true negative; CD, case detected; ICER incremental cost-effect ratio, MLPA, Multiplex ligation probe amplification; PCR, polymerase chain reaction; RT-PCR, real-time PCR; CISH, chromogenic in situ hybridisation; SNP, single nucleotide polymorphism; NGS, next generation sequencing.
